# Deletion of junctional adhesion molecule A from platelets increases early‐stage neointima formation after wire injury in hyperlipidemic mice

**DOI:** 10.1111/jcmm.13083

**Published:** 2017-02-17

**Authors:** Zhen Zhao, Tanja Vajen, Ela Karshovska, Annemiek Dickhout, Martin M. Schmitt, Remco T.A. Megens, Philipp von Hundelshausen, Thomas A. Koeppel, Tilman M. Hackeng, Christian Weber, Rory R. Koenen

**Affiliations:** ^1^ Institute for Cardiovascular Prevention (IPEK) Ludwig‐Maximilians‐University Munich Munich Germany; ^2^ Division of Vascular and Endovascular Surgery Ludwig‐Maximilians‐University Munich Munich Germany; ^3^ Cardiovascular Research Institute Maastricht (CARIM) Maastricht University Maastricht The Netherlands; ^4^ German Centre for Cardiovascular Research (DZHK) partner site Munich Heart Alliance Munich Germany

**Keywords:** neointima, junctional adhesion molecule, platelet, leucocyte

## Abstract

Platelets play an important role in the pathogenesis of vascular remodelling after injury. Junctional adhesion molecule A (JAM‐A) was recently described to regulate platelet activation. Specific deletion of JAM‐A from platelets resulted in increased reactivity and in accelerated progression of atherosclerosis. The aim of this study was to investigate the specific contribution of platelet‐derived JAM‐A to neointima formation after vascular injury. Mice with or without platelet‐specific (tr)JAM‐A‐deficiency in an apolipoprotein e (apoe^−/−^) background underwent wire‐induced injury of the common carotid artery. *Ex vivo* imaging by two‐photon microscopy revealed increased platelet coverage at the site of injury in trJAM‐A‐deficient mice. Cell recruitment assays showed increased adhesion of monocytic cells to activated JAM‐A‐deficient platelets than to control platelets. Inhibition of α_M_β_2_ or GPIbα, but not of CD62P, suppressed those differences. Up to 4 weeks after wire injury, intimal neoplasia and neointimal cellular content were analysed. Neointimal lesion area was increased in trJAM‐A^−/−^ apoe^−/−^ mice and the lesions showed an increased macrophage accumulation and proliferating smooth muscle cells compared with trJAM‐A^+/+^ apoe^−/−^ littermates 2 weeks, but not 4 weeks after injury. Re‐endothelialization was decreased in trJAM‐A^−/−^ apoe^−/−^ mice compared with controls 2 weeks after injury, yet it was complete in both groups after 4 weeks. A platelet gain of function by deletion of JAM‐A accelerates neointima formation only during earlier phases after vascular injury, through an increased recruitment of mononuclear cells. Thus, the contribution of platelets might become less important when neointima formation progresses to later stages.

## Introduction

Junctional adhesion molecule A (JAM‐A, F11 receptor, CD321) is a member of the immunoglobulin superfamily of transmembrane adhesion molecules 1. In epi‐ and endothelial cells, JAM‐A typically locates at intercellular junctions, where it maintains cell layer permeability through homophilic interactions 2, 3. However, during inflammatory conditions, JAM‐A is relocated to the apical endothelial cell surface and may thereby also facilitate leucocyte recruitment by heterophilic interactions with the leucocyte α_L_β_2_ integrin 4, 5, 6, 7. This process was shown to be important for vascular remodelling, as genetic deletion of JAM‐A reduced monocyte recruitment and neointima formation after vascular injury 8. Interestingly, whereas somatic deletion of JAM‐A did not affect the development of atherosclerosis, a conditional knockdown of JAM‐A in endothelial cells resulted in reduction in plaque formation, which could be explained by a decreased recruitment of monocytes to atherosclerotic predilection sites 9. JAM‐A was initially identified on platelets as the antigen for F11, a monoclonal antibody that resulted in platelet activation through crosslinking with the FcγRII receptor 10. Later studies demonstrated that JAM‐A associates with integrin α_IIb_β_3_ and serves as an endogenous inhibitor of platelet outside‐in signalling 11. Thus, deletion of JAM‐A from platelets leads to a lower activation threshold by soluble and immobilized agonists, resulting in increased laser‐induced thrombus formation in mice 12. This resulting platelet hyperreactivity accelerated plaque formation in a mouse model of atherosclerosis, particularly in earlier stages of the disease 13. As platelets play an important role in vascular remodelling after injury 14, we aimed to investigate the role of JAM‐A in platelets in a mouse model of wire‐induced neointima formation.

## Materials and methods

### Mice

Mice carrying cre recombinase under the control of the platelet factor 4 (PF4) promoter 15 were backcrossed with apolipoprotein e‐deficient (apoe^−/−^) mice (C57Bl/6) for more than 10 generations. These mice were crossed with JAM‐A^flox/flox^ apoe^−/−^ mice to generate platelet‐specific JAM‐A‐deficient (trJAM‐A^−/−^ apoe^−/−^) mice. Mice not carrying PF4‐cre recombinase (trJAM‐A^+/+^ apoe^−/−^ mice) were used as controls 13. The mice were bred under specific pathogen free conditions at the animal facilities of the LMU Munich and at InnoSer BV, Lelystad, the Netherlands. All animal experiments were approved by local authorities (Regierung von Oberbayern, Munich, Germany, and the animal ethical committee at Maastricht University, Maastricht, the Netherlands).

### Mouse wire injury model

In this model, the endothelium of the mouse carotid artery was completely denuded using an angioplasty catheter guide wire without damaging the internal lamina 16, 17, 18. Six‐ to eight‐week‐old mice were put on a high‐fat diet (HFD, 21% fat, 19.5% casein, 0.15% cholesterol, ssniff, Soest, Germany) for 1 week before until 2 or 4 weeks after wire injury. After mouse anaesthesia (ketamine 80 mg/kg and medetomidine 0.3 mg/kg), the left carotid artery and branches were exposed under a stereomicroscope (Stemi: 2000‐c linked with the KL 1500 LCD; Zeiss Oberkochen, Germany) by midline neck incision; 7/0 surgical suture (IO 051391; Serag Wiessner, Naila,Germany.) was looped around the distal left common carotid artery and proximal internal and external carotid arteries for temporary control of blood flow. An additional suture was looped around distal external carotid artery near the bifurcation. After crosscutting a small hole on the external artery between those two sutures around the external artery, a flexible angioplasty guide wire with 0.36 mm diameter was inserted into the common carotid artery and advanced for approx. 1 cm. Complete and uniform endothelial denudation was achieved by three passes along the common carotid artery with a rotating motion. Following removal of the wire, the proximal and distal sutures around the external carotid artery were tied off and the blood flow was restored, which was monitored by vessel pulsation and the colour of blood. The skin incision was closed by two suture clips (BN507R; Aesculap Tuttlingen, Germany). After operation, the ketamine was antagonized (atipamezole hydrochloride, 0.3 mg/kg) for recovery and the mice received meloxicam (0.2 mg/kg) as analgetic.

### Platelet aggregation measurement

Mice were anaesthetized and blood was retro‐orbitally collected into EDTA‐coated tubes (41.1504.005; Sarstedt Nümbrecht, Germany). Platelet aggregation assessment (multiple electrode aggregometry, MEA) was performed within 2 hrs after platelet isolation. Platelet aggregation was assessed in a Multiplate^®^ platelet analyser according to manufacturer's instructions (Roche Diagnostics, Mannheim, Germany) 13. Blood samples were pre‐incubated with 0.9% saline (1:1) at 37°C for 3 min. without stirring. Collagen (2 μg/ml, 3 μg/ml or 4 μg/ml, cat nr. 900004; Loxo Dossenheim, Germany) was added and the measurement of electrical impedance was continuously recorded for 5 min. under stirring. The cumulative aggregation values of platelet aggregation were expressed as ‘aggregation units’ in 5 min. (AU*min).

### Platelet activation measurement by flow cytometry

Platelets were isolated from C57Bl/6, trJAM‐A^+/+^, trJAM‐A^−/−^ mice as previously described 13 and resuspended at a concentration of 5 × 10^7^/ml in Tyrode Hepes (TH) buffer (136 mM NaCl, 5 mM Hepes, 0.42 mM NaH_2_PO_4_, 2.7 mM KCl, 2 mM MgCl_2_, 0.1% glucose, 0.1% BSA pH 7.4). Platelets were stimulated with 0, 0.1, 0.5, 1.0, 2.5, 4.0 and 10 nM human α‐thrombin and stained with an anti‐CD62P‐APC‐labelled antibody (clone Psel.KO2.3) or isotype control in the presence of 2 mM CaCl_2_. Following incubation of 15 min. at room temperature, CD62P expression was analysed with a flow cytometer (BD Accuri Cytometer Franklin Lakes, New Jersey, USA).

### Two‐photon laser scanning microscopy of injured carotid artery

Two‐photon laser scanning microscopy (TPLSM) was used to image adherent platelets in explanted wire‐injured carotid arteries 9. The trJAM‐A^+/+^apoe^−/−^ and trJAM‐A^−/−^apoe^−/−^ mice (*n* = 5 per group) were fed a HFD for 1 week before wire injury as described above. One hour after operation, the injured carotid artery was explanted, mounted in a customized perfusion chamber and pressurized at 80 mmHg. Next, antibodies diluted in HBSS were injected into artery lumen and incubated for 30 min. at room temperature. The complete denudation of the endothelium was assessed by labelling the endothelium by an anti‐CD31 Alexa Fluor^®^ (AF) 488‐coupled antibody (5 μg/ml, 102414; eBioscience Heidelberg, Germany). Platelets present on the denudated artery wall were stained with an anti‐CD41 PE‐coupled antibody (3.5 μg/ml, 558040; BD Pharmingen Heidelberg, Germany.). TPLSM was performed with a Leica SP5IIMP system coupled to a 20xNA1.00 objective and a Ti:Sa pulsed laser (Maitai DeepSee; Spectra Physics, Santa Clara, CA, USA) for three‐dimensional (3D) imaging of adherent platelets on the injured artery wall. The emitted fluorescent signals were simultaneously detected by hybrid diode detectors tuned for the corresponding wavelengths using an acousto‐optical beam splitter: 510–540 nm (AF488) and 570–600 nm (PE). Z‐stacks were acquired at 0.1 Hz including twofold frame averaging. One field of view (FOV = 330 μm^2^; voxel size: 0.32 × 0.32 × 1 μm^3^) was recorded. Each image was acquired using LAS AF 2.0 software (Leica, Mannheim, Germany) and processed using Image Pro Premier 9.1 software (Media Cybernetics, Rockville, MD, USA). More than three FOVs without endothelial signals in each injured artery were constructed as 3D pictures and platelet volumes were automatically calculated. The values were expressed as mean ± S.D. (μm^3^).

### Attachment and detachment assays under flow

Platelets were isolated from C57Bl/6, trJAM‐A^+/+^, trJAM‐A^−/−^ mice (immobilization on collagen) and from trJAM‐A^+/+^apoe^−/−^ and trJAM‐A^−/−^apoe^−/−^ mice (immobilization on APTES) as previously described 13 and resuspended at a concentration of 2 × 10^7^/ml in TH buffer. Washed platelets were immobilized on 3‐aminopropyltriethoxysilane (APTES)‐ or soluble rat‐tail collagen‐treated (30 μg/ml) glass coverslips (Menzel, 24 × 60 mm, #1.5) for 1.5 hrs at 37°C in a moisture chamber. Nonspecific binding was blocked with 0.5% human serum albumin (HSA) in HEPES buffer for 30 min. at 37°C 19. A confluent layer of spread platelets was formed, which was examined by phase contrast microscopy (TE‐2000‐E; Nikon Melville, NY, USA) before and after flow perfusion and was not affected by flow shear stress. RAW264.7 cells (mouse monocyte/macrophage, ATCC^®^ TIB‐71^™^) were cultured in Dulbecco′s modified Eagle′s medium (DMEM, 41965‐039; Gibco Waltham, MA,USA), which was modified to contain 10%FBS, 1 mM sodium pyruvate, 1500 mg/l sodium bicarbonate and 0.1% gentamicin, and THP1 cells (human monocyte, DSMZ, ACC‐16) were cultured in RPMI medium supplemented with 10% FCS and 1% penicillin and streptomycin at 37°C. Monocytic cells were resuspended at 10^6^/ml in HBSS with 10 mM HEPES (pH 7.4) and 0.2% HSA (HHHSA buffer) and labelled with 1 μM Syto‐13 (Thermofisher Scientific, Waltham, MA, USA). Platelet‐coated glass coverslips were assembled in parallel flow chambers (0.4‐mm Luer sticky slides, ibidi^®^, Martinsried, Germany) and then mounted in a phase contrast (Nikon TE‐2000‐E) using a 10× objective or a fluorescent microscope (EVOS‐FL) using a 20× objective and a CCD camera. Platelets were activated by perfusion of thrombin (0.5 nM for collagen and 1 nM for APTES) in HHHSA buffer for 3 min. at 37°C, followed by perfusion of HHHSA buffer for 3 min. RAW267.4 cells and platelets were pre‐incubated with 60 μM eptifibatide to inhibit integrin α_IIb_β_3_ (Integrilin, GlaxoSmithKline Brentford, Middlesex, UK.), 20 μM CD62P inhibitor (2748/10, Tocris Biochemicals), 2.5 μg/ml CD42b (GPIbα) depletion antibody (R300, emfret analytics) or 20 μg/ml CD11b (integrin αM) antibody (antimouse: 101201, Biolegend or anti‐human: CBRM1/29), or equal concentrations of isotype control antibody, respectively. Prior to RAW264.7 cell perfusion, 1 mM MgCl_2_ and 1 mM CaCl_2_ was added into the cell suspensions and perfused for 3 min. at a shear stress of 0.2 dynes/cm^2^. Following 3 min. of perfusion, the number of firmly adherent cells per mm^2^ (no movement for >10 sec.) was counted within 3 min. In the experiments using platelets immobilized on APTES, the number of tethering cells (bound for less than 10 sec.) was also determined within the same three‐min period 19. For measurement of flow‐dependent detachment, platelets immobilized on a collagen‐coated glass coverslip were assembled in an Ibidi^™^ microslide chamber (with a chamber height of 0.1 mm for RAW264.7 and 0.2 for THP1 cells) and were subsequently perfused without or with a thrombin (0.5 nM) and treated without or with antibodies as described above, prior to perfusion with monocytic cells (at 0.2 dynes/cm^2^). After 5 min., shear stress was incrementally increased from 0 to 20 dynes/cm^2^ every 30 sec. and cells were counted in three different fields after each increment.

### Histological analysis of neointima formation

Neointima formation was induced in six‐ to eight‐week‐old male and female mice (2 weeks, *n* = 9–10; 4 weeks *n* = 11–13) by feeding a high‐fat diet (HFD, 21% fat, 19.5% casein, 0.15% cholesterol, ssniff, Soest, Germany) for 1 week before and 2 or 4 weeks after carotid wire injury. Mice were anaesthetized as described earlier and the injured carotid arteries were dissected after whole body *in situ* perfusion with 4% buffered formaldehyde (PFA) (Carl Roth, Karlsruhe, Germany). Following fixation in 4% PFA for 3–4 hrs, carotid arteries were dehydrated and embedded in paraffin (Leica, ASP200S and EG1160). The injured common carotid arteries were cut in sequential sections (4 μm thick) using a sliding microtome (Leica, RM2155). Ten sections per mouse within a standardized distance from the bifurcation (400 μm) were analysed.

After deparaffinization and rehydration, sections were stained with Elastic van Gieson (EVG) (Baacklab, Germany) and covered with mounting solution (Roti‐HistoKit II, Carl Roth Karlsruhe, Germany.). Neointima formation was evaluated using a light microscope (Leica DM RBE), connected to a DFC425C camera. The area of neointima formation was calculated by subtracting the lumen area from the area enclosed by the internal elastic lamina using Leica software (Leica Application Suite V4.6). Immunofluorescence staining of adjacent sections was performed to evaluate the neointima cellular content in particular macrophages (MAC2), smooth muscle cells (α‐Actin), proliferating cells (Ki67) and endothelium (von Willebrand, VWF). After deparaffinization, rehydration, heat‐induced antigen retrieval (Dako, Hamburg, Germany) and blocking of unspecific protein binding by 10% goat serum (Dako, X0907) for 20 min., carotid arteries sections were incubated with primary antibodies at 4°C overnight. Primary antibodies: anti‐MAC‐2 (2.5 μg/ml, Cedarlane, CL8942AP), anti‐α‐smooth muscle actin (0.7 μg/ml, Dako, M0851), anti‐Ki67 antibodies (3 μg/ml, Abcam, ab15580) and anti‐VWF (5.5 μg/ml, Dako, A0082). DyLight^®^‐488‐ and DyLight^®^‐550‐conjugated secondary antibodies were used for detection (all Abcam, Cambridge, UK). Fluorescent images were recorded with a DM 6000B fluorescence microscope (Leica, Solms, Germany), connected to a monochrome digital camera (DFC 365FX). Macrophages were quantified as MAC2‐positive area within the neointima area by using colour threshold measurements according to fluorescence intensity (Leica, LAS V4.6). Smooth muscle cell (SMC) content was expressed as per cent α‐actin‐positive cells of all neointima cells. Proliferating SMC were counted as α‐actin and Ki67 double‐positive cells that have a DAPI‐positive nucleus. Re‐endothelialization was indicated by VWF‐positive length in lumen circumference.

### Statistics

Statistics were performed with Prism 5.0 (GraphPad Software). San Diego, California, USA. Data were analysed by two‐way anova with Tukey's post‐test or unpaired *t*‐test or Wilcoxon–Mann–Whitney test to evaluate two‐tailed levels of significance. Differences with *P *<* *0.05 were considered as statistically significant.

## Results

**Figure 1:**
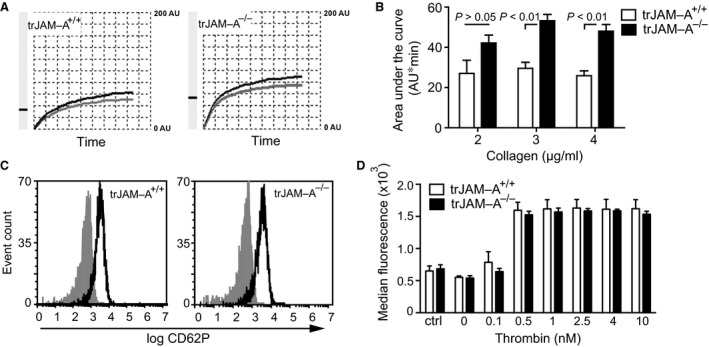
trJAM‐A deficiency increases platelet aggregation. Aggregation of platelets from JAM‐A^+/+^ and JAM‐A^−/−^ mice in response to collagen (3 μg/ml) is shown as representative tracings (**A**) and quantified as area under the curve (AU*min) (**B**). Activation of JAM‐A^+/+^ and JAM‐A^−/−^ platelets after stimulation with thrombin at indicated concentrations as measured by CD62P surface exposure by flow cytometry. (**C**) Representative histograms before (solid grey) and after (black line) stimulation with 0.5 nM of thrombin. (**D**) Median fluorescence intensity of CD62P surface exposure after thrombin stimulation. Ctrl is isotype control and 10 nM of thrombin. *P* values were calculated by anova with Tukey's post‐test (*n* = 8–15).

**Figure 2:**
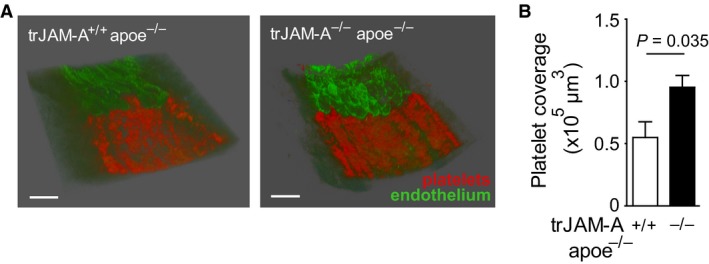
trJAM‐A deficiency increases platelet adhesion after vascular injury. Three‐dimensional (3D) luminal view of the vessel wall recorded with two‐photon laser scanning microscopy (**A**) shows platelets (CD41^+^, red) adherent to endothelium (CD31^+^, green) at the border of the arterial injury (1 hr after denudation). Platelet coverage (μm^3^) was quantified at sites of complete denudation (**B**). *P* values were calculated by unpaired two‐tailed *t*‐test without Welch correction (**B**,* n* = 5). Scale bar: 50 μm.

**Figure 3:**
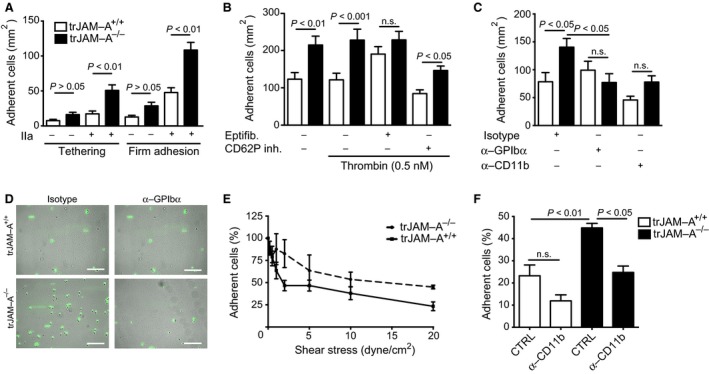
trJAM‐A deficiency promotes flow‐resistant monocytic cell adhesion. Transient tethering and stable interactions of RAW264.7 cells with platelets from JAM‐A^+/+^ apoe^−/−^ and JAM‐A^−/−^apoe^−/−^ mice immobilized on APTES‐coated glass slides under flow conditions, without or with thrombin activation (IIa, 1 nM) (**A**). Stable interactions of RAW264.7 cells with platelets from JAM‐A^+/+^ and JAM‐A^−/−^ mice immobilized on collagen‐coated glass slides under flow conditions, without or with thrombin activation (IIa, 0.5 nM) in the presence of indicated inhibitors (**B**,** C**). Representative micrographs of RAW264.7 cells adherent to JAM‐A^+/+^ and JAM‐A^−/−^ platelets after treatment with isotype control or anti‐GPIbα antibodies (**D**). Cell adhesion expressed as percentage of initially adherent cells under incrementally increased shear stress (dyne/cm^2^) on immobilized platelets after thrombin activation (IIa, 0.5 nM) (**E**). Adherent cells (%) on thrombin‐activated platelets at a shear stress of 20 dyne/cm^2^ in the presence of blocking anti‐CD11b antibodies (**F**). *P* values were calculated by anova with Tukey's or Bonferroni's post‐test (**B**,** C**,** F** *n* = 3–5). Scale bar: 100 μm.

Platelet‐specific JAM‐A (trJAM‐A^−/−^) mice and control littermates (trJAM‐A^+/+^) in an apolipoprotein e‐deficient (apoe^−/−^) background were generated in our previous study 13. The previously observed elevated reactivity of JAM‐A^−/−^ platelets was confirmed by stimulation of whole blood from trJAM‐A^−/−^ and trJAM‐A^+/+^ mice by fibrillar collagen, a major matrix component exposed upon vascular injury (Fig. 1 A, B), supporting the role of JAM‐A as an endogenous platelet activation inhibitor 11, 12, 13. On the other hand, no differences in CD62P (P‐selectin) expression were observed upon activation of isolated platelets from trJAM‐A^−/−^ and trJAM‐A^+/+^ with increasing amounts of thrombin (Fig. 1C and D). To investigate the functional consequences of platelet‐specific JAM‐A deficiency for neointima formation, a model of wire‐induced arterial injury was implemented 8. Endothelial denudation of the common carotid artery caused immediate platelet coverage on the injured arterial wall, which was enhanced by platelet‐JAM‐A deficiency, 1 hr after wire injury, as visualized by *ex vivo* two‐photon laser scanning microscopy (TPLSM) 7 (Fig. 2 A, B). As a result, activated platelets adherent on the injured arterial wall may promote neointima formation through the attraction of leucocytes and by the release of degranulation products that stimulate SMC proliferation 20. Moreover, platelets may form a bridge between monocytes and the vessel wall during vascular injury 14, 21. Thus, the ability of surface‐adherent JAM‐A^+/+^ and JAM‐A^−/−^ platelets to attract monocytic RAW264.7 cells was investigated under flow conditions. Interestingly, JAM‐A‐deficient platelets immobilized onto APTES‐coated surfaces were able to recruit RAW264.7 cells more efficiently than JAM‐A^+/+^ control platelets, most notably after stimulation with thrombin (1 nM), on the level of both transient tethering and stable adhesion (Fig. 3A). An increased flow‐resistant recruitment of RAW264.7 cells to JAM‐A^−/−^ platelets was also observed when platelets were immobilized onto collagen‐coated surfaces (Fig. 3B and Fig. S1A). However, activation with thrombin did not lead to a further increase in RAW264.7 recruitment, hinting towards maximal platelet activation under those conditions (Fig. 3B). Surprisingly, treatment of the adherent platelets with the α_IIb_β_3_ antagonist eptifibatide resulted in an increase in RAW264.7 adhesion to JAM‐A^+/+^ platelets, which might be due to the induction of activation epitopes in α_IIb_β_3_ by this antagonist 22. Inhibition of CD62P, which mediates direct leucocyte–platelet contacts, slightly decreased the recruitment of RAW264.7 cells to adherent platelets, but did not abolish the increased mononuclear cell recruitment by JAM‐A^−/−^ platelets (Fig. 3B). Monocyte adhesion can also occur through interactions of the integrin α_M_β_2_ (Mac‐1) with GPIbα (CD42b) or JAM‐C on platelets 14, 23. The enhanced recruitment of RAW264.7 cells to JAM‐A^−/−^ platelets was significantly reduced when either α_M_β_2_ or GPIbα were inhibited by blocking antibodies (Fig. 3C and D). In addition, significant differences in mononuclear cell recruitment between immobilized JAM‐A^+/+^ and JAM‐A^−/−^ platelets were no longer observed. To investigate the shear stress dependence of platelet monocytic cell interactions, cell detachment assays were performed. Interestingly, the adhesion of RAW264.7 cells on JAM‐A^−/−^ platelets was more resistant to shear stress than the binding of these cells to JAM‐A^+/+^ control platelets (Fig. 3E). At a shear stress of 20 dynes/cm^2^, almost half of the initially attached RAW264.7 cells still adhered to the JAM‐A^−/−^ platelets (45 ± 3.6%), which is nearly twice the cells that bound to JAM‐A^+/+^ platelets (23 ± 8.5%). The binding was reduced after treatment with antibodies to CD11b (Fig. 3F). Similar findings were obtained for THP1 cells on human platelets, although the interactions were somewhat less shear resistant. (Fig. S1B, C).

**Figure 4:**
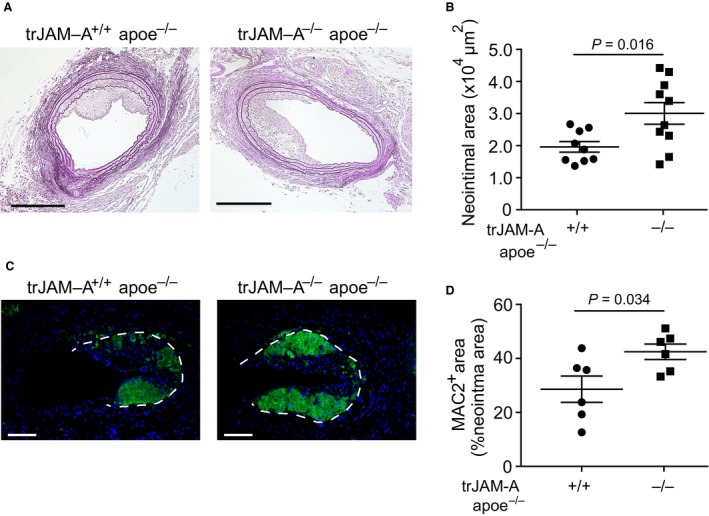
trJAM‐A deficiency increases neointima formation and plaque macrophage content. Neointima 2 weeks after wire injury was visualized by EVG staining (**A**) and quantification (**B**) of neointimal area (μm^2^). Immunofluorescence of neointimal macrophages (**C**, MAC2^+^, green) and quantification (**D**). *P* values were calculated by unpaired two‐tailed *t*‐test with (**B**,* n* = 9–10) or without (**D**,* n* = 6) Welch correction. Scale bars: 200 μm (**A**) and 100 μm (**C**).

**Figure 5:**
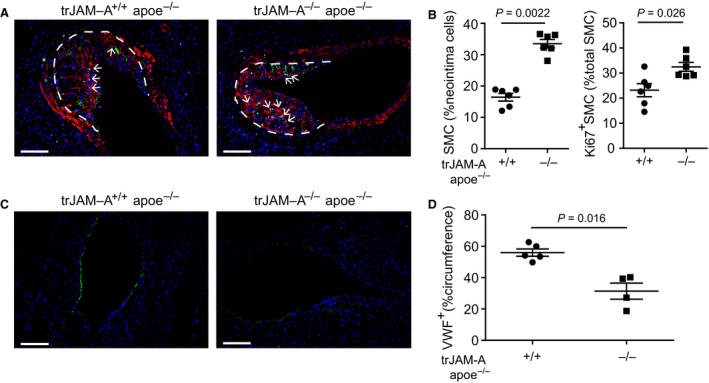
Cellular composition of neointima from trJAM‐A^+/+^ apoe^−/−^ and trJAM‐A^−/−^ apoe^−/−^ mice, 2 weeks after wire injury. Immunofluorescence of SMC (**A**, α‐Actin^+^, red) and proliferating cells (**A**, Ki67^+^, green) and their quantification (**B**, SMC, left panel and Ki67^+^ SMC, right panel). Proliferating SMC were marked with arrows (**A**, α‐Actin^+^, Ki67^+^ and DAPI ^+^). Endothelium was visualized by staining of VWF (green) (**C**), and length of VWF ^+^ lining was expressed as percentage of luminal circumference (**D**). *P* values were calculated by Mann–Whitney *U*‐test (**B**,* n* = 6 and **D**,* n* = 4–5). Neointima areas were demarcated with dashed lines. Nuclei were stained with DAPI (blue). Scale bars: 100 μm.

As platelets contribute to neointima formation through the attraction of leucocytes to the site of injury 14, 24, we investigated a role of JAM‐A‐deficient platelets in arterial remodelling. Neointima formation was investigated in trJAM‐A^+/+^ apoe^−/−^ and trJAM‐A^−/−^ apoe^−/−^ mice 2 and 4 weeks after wire injury. Platelet‐specific JAM‐A deficiency led to an increase in neointima formation 2 weeks, but not 4 weeks after injury (Fig. 4 A, B and Fig. S2 A, B). Quantification of neointimal cell composition revealed an increased number of MAC2‐positive macrophages in trJAM‐A^−/−^ apoe^−/−^ mice, 2 weeks after wire injury, compared with the control group (Fig. 4 C and D). These findings are consistent with our previous observations that JAM‐A‐deficient platelets facilitate macrophage accumulation in developing atherosclerotic plaques 13. In addition, the content of SMC was notably increased in platelet‐JAM‐A‐deficient mice as compared to controls (Fig. 5 A, B left). The increase in SMC content might be due to enhanced proliferation, as the percentage SMC carrying the Ki67 proliferation marker was markedly increased in trJAM‐A^−/−^ apoe^−/−^ mice (Fig. 5 A, B right). Furthermore, re‐endothelialization was delayed in mice with platelet‐specific JAM‐A deficiency, as shown by quantification of the endothelial marker VWF, 2 weeks after injury (Fig. 5 C and D). However, cellular composition of the neointimal lesions did not differ between trJAM‐A^+/+^ apoe^−/−^ and trJAM‐A^−/−^ apoe^−/−^ mice, 4 weeks after injury (Fig. S2 C–H).

## Discussion

In the current study, we further characterize the function of JAM‐A on platelets in vascular remodelling and observed that platelet‐specific deletion of JAM‐A led to increased reactivity of platelets, enhanced coverage of platelets at the site of injury and acceleration of early‐stage neointima formation. Platelets may contribute to neointima formation through the attraction of leucocytes to the site of injury 24 and by the release of growth factors and mitogens, stimulating the proliferation of SMC 20. In our model experiments, we could demonstrate that immobilized activated JAM‐A‐deficient platelets attracted monocytic cells more efficiently than control platelets, both when immobilized to APTES‐ or to collagen‐coated surfaces. This is consistent with previous findings, where JAM‐A‐deficient platelets more avidly interacted with monocytes 13. Platelet–leucocyte interaction is mediated through several surface receptors, for example CD62P 25, GPIbα 14 and α_IIb_β_3_ 19, 26. Interestingly, blockade of CD62P reduced the overall recruitment of RAW264.7 cells, yet the differences between JAM‐A^+/+^ and JAM‐A^−/−^ platelets remained. This is in line with the similar CD62P surface expression profiles of JAM‐A^+/+^ and JAM‐A^−/−^ platelets in response to increasing concentrations of thrombin. Unlike platelet aggregation, CD62P release appears to be less affected by the hyper‐reactive phenotype of JAM‐A‐deficient platelets. This also indicates that deficiency of JAM‐A does not influence CD62P‐mediated leucocyte recruitment to adherent platelets. Inhibition of α_IIb_β_3_ by tirofiban was shown to reduce leucocyte recruitment to adherent platelets in our previous studies and to abolish the difference between JAM‐A‐deficient and wild‐type platelets 13. However, antagonism of α_IIb_β_3_ by eptifibatide implemented in this study did not affect the recruitment of RAW264.7 cells to JAM‐A^−/−^ platelets yet increased the arrest of those cells to JAM‐A^+/+^ platelets. A possible explanation might be that synthetic RGD mimics such as eptifibatide can induce integrin activation 22, potentially leading to an increased recruitment of monocytic cells through the α_M_β_2_–fibrinogen–α_IIb_β_3_ axis 19. Blockade of GPIbα, however, did abolish the difference between the JAM‐A^−/−^ and JAM‐A^+/+^ genotypes. In addition, inhibition of the counter receptor for GPIbα on RAW264.7 cells, α_M_β_2_, resulted in a marked decrease in cell adhesion and also reduced the increased ability of JAM‐A‐deficient platelets to recruit monocytic cells. Thus, we have identified a role for the GPIbα–α_M_β_2_ axis in the enhanced monocyte recruitment by JAM‐A‐deficient platelets. At present, the underlying mechanisms for this observation are unclear. As collagen can induce strong platelet activation through the GPVI receptor 27, it is possible that this activation pathway is also influenced by JAM‐A. However, additional evidence is required to support this notion. It should be noted that the shear stress levels in some of our *in vitro* model systems are considerably lower than those present in the carotid artery 28. Unlike the interaction of GPIbα with VWF, which can mediate platelet adhesion only at high shear rates of >6000 per s, the binding of GPIbα to α_M_β_2_ can occur also at low shear rates. This is because the I‐domain of α_M_β_2_ requires stimulation by cellular signal transduction rather than by mechanical activation at high shear rates to become active. Yet, the interaction of GPIbα with α_M_β_2_ has also been shown to support monocyte binding to adherent platelets under arterial flow conditions 29 and specific inhibition of this interaction reduced neointima formation after arterial injury 14. This is also supported by our findings, where blockade of CD11b reduced platelet‐monocytic cell interaction at higher shear stress regimes. Interestingly, monocytes adherent to JAM‐A‐deficient platelets were less prone to detach at higher shear stress. It remains a subject for future investigation whether this is due to a regulation of platelet integrins by JAM‐A 12, or by an inhibitory influence of platelet‐JAM‐A on leucocyte integrins *in trans*, analogous to that observed for developmental endothelial locus 1 (Del‐1) 30. The overall increased recruitment of monocytes is also reflected by the elevated content of macrophages in the neointimal lesions of the trJAM‐A^−/−^ apoe^−/−^ mice, 2 weeks after injury. Given the observed enhanced coverage of JAM‐A‐deficient platelets on the newly inflicted vascular injury and the improved ability of those platelets to attract monocytic cells, we hypothesize that the elevated macrophage count found in the lesions of trJAM‐A^−/−^ apoe^−/−^ mice is a combined result of those effects. However, an increased content of SMC was also observed in the neointimal lesions, 2 weeks after injury. This elevated content was also accompanied by a higher fraction of Ki67‐positive SMC, indicative of increased proliferation. We have previously demonstrated that plasma levels of the chemokine CXCL4 (platelet factor 4) were increased in trJAM‐A^−/−^ apoe^−/−^ mice on a high‐fat diet, possibly to due to an increased release by JAM‐A‐deficient platelets 13. This notion is supported by a recent study that identified CXCL4 as an important mediator of the platelet‐driven injury response of SMC 31. An increased release of CXCL4 might also explain the delayed re‐endothelialization in trJAM‐A^−/−^ apoe^−/−^ mice, as this chemokine is an established inhibitor of endothelial proliferation 32.

It might be surprising that the pronounced effects of platelet‐specific JAM‐A deficiency observed in earlier stage neointima formation disappeared at later stages. Previous work has highlighted a context‐ and cell type‐dependent role of JAM‐A as a mediator of inflammatory leucocyte recruitment 3, 9, 33. Thus, the effects of the platelet‐specific JAM‐A deficiency might be counteracted by other mechanisms during the course of neointima formation. Similar results were observed in a model of diet‐induced atherosclerosis, where the consequences of platelet‐specific JAM‐A deficiency were most obvious during early stages of atherosclerosis 13. Besides, re‐endothelization is complete at later stages and the newly covered endothelial lining would inhibit platelet adhesion, thereby lowering the contribution of platelets to the process of vascular remodelling, compared with the initial stage of vascular injury. Further, the multiple roles of JAM‐A in the diverse cell types led to different effects on the manifestation of diet‐induced atherosclerosis. Bone marrow‐ and platelet‐specific deletion of JAM‐A increased plaque formation, whereas endothelium‐specific deletion reduced plaque burden in apoe^−/−^ mice 9, 13. Total somatic deletion of JAM‐A did not affect plaque formation 9. The diverse cellular roles of JAM‐A might also explain why a somatic deletion of JAM‐A led to a net decrease in the advanced neointima in our previous study 8. As JAM‐A‐deficient leucocytes are characterized by defective transendothelial migration 9, 33, the net process of neointima formation in the somatic JAM‐A^−/−^ mice might be hampered by the defective leucocyte recruitment, which is not compensated by a gain of function in platelet reactivity. According to our current and previous findings, the effects of platelet‐specific JAM‐A deficiency and the associated gain of function might be most pronounced during initial stages of vascular remodelling and decline at advanced stages.

Taken together, we have demonstrated that specific deletion of JAM‐A in platelets leads to an increase in early‐stage neointima formation, possibly by an increased attraction of monocytes and the stimulation of SMC proliferation. This study further emphasizes the role of platelets in vascular remodelling and provides an additional example of the multifaceted cell type‐specific role of JAM‐A in vascular inflammation. As in‐stent thrombosis and restenosis are still a clinical challenge 34, precise knowledge of the underlying molecular processes, such as the involvement of platelets, is required for the development of more effective therapeutics.

## Author contributions

Z.Z., T.V., E.K. and A.D. performed and analysed experiments, and Z.Z. partially wrote the manuscript, M.M.S. and R.T.A.M performed imaging, P.v.H. provided intellectual and material input, T.A.K. provided necessary resources, T.M.H. provided intellectual input, C.W. raised funding and provided intellectual input, and R.R.K. supervised study, analysed data, obtained funding and wrote the manuscript.

## Funding sources

This work was supported by Deutsche Forschungsgemeinschaft (DFG Ko2948/1‐2 and by FOR809 and SFB1123/A1 and to C.W., SFB1123/A2 to R.R.K., SFB1123/Z1 to R.T.A.M.), the DFG International Research Training Group GRK1508 (EuCAR), and the Netherlands Foundation for Scientific Research (ZonMW VIDI 016.126.358 awarded to R.R.K). The Leica two‐photon laser scanning microscope was supported by the DFG (INST409/97‐1) and LMU. In addition, the study was partly supported by the European Research Council (ERC Advanced Grant 249929 awarded to C.W.).

## Conflicts of interest

The authors declare no conflicts of interest.

## Supporting information


**Figure S1** Adhesion of monocytic cells to immobilized platelets

**Figure S2** Neointima area and composition in trJAM‐A^+/+^ apoe^−/−^ and trJAM‐A^−/−^ apoe^−/−^ mice, 4 weeks after wire‐injury
