# Supplementary material for: Deletion of junctional adhesion molecule A from platelets increases early‐stage neointima formation after wire injury in hyperlipidemic mice
Source: J Cell Mol Med. 2017 Feb 17;21(8):1523–31. doi: 10.1111/jcmm.13083 (PMC5542900; doi:10.1111/jcmm.13083)
Supplement: Supplementary file 1 — Figure S1 Adhesion of monocytic cells to immobilized platelets [file JCMM-21-1523-s001.docx]

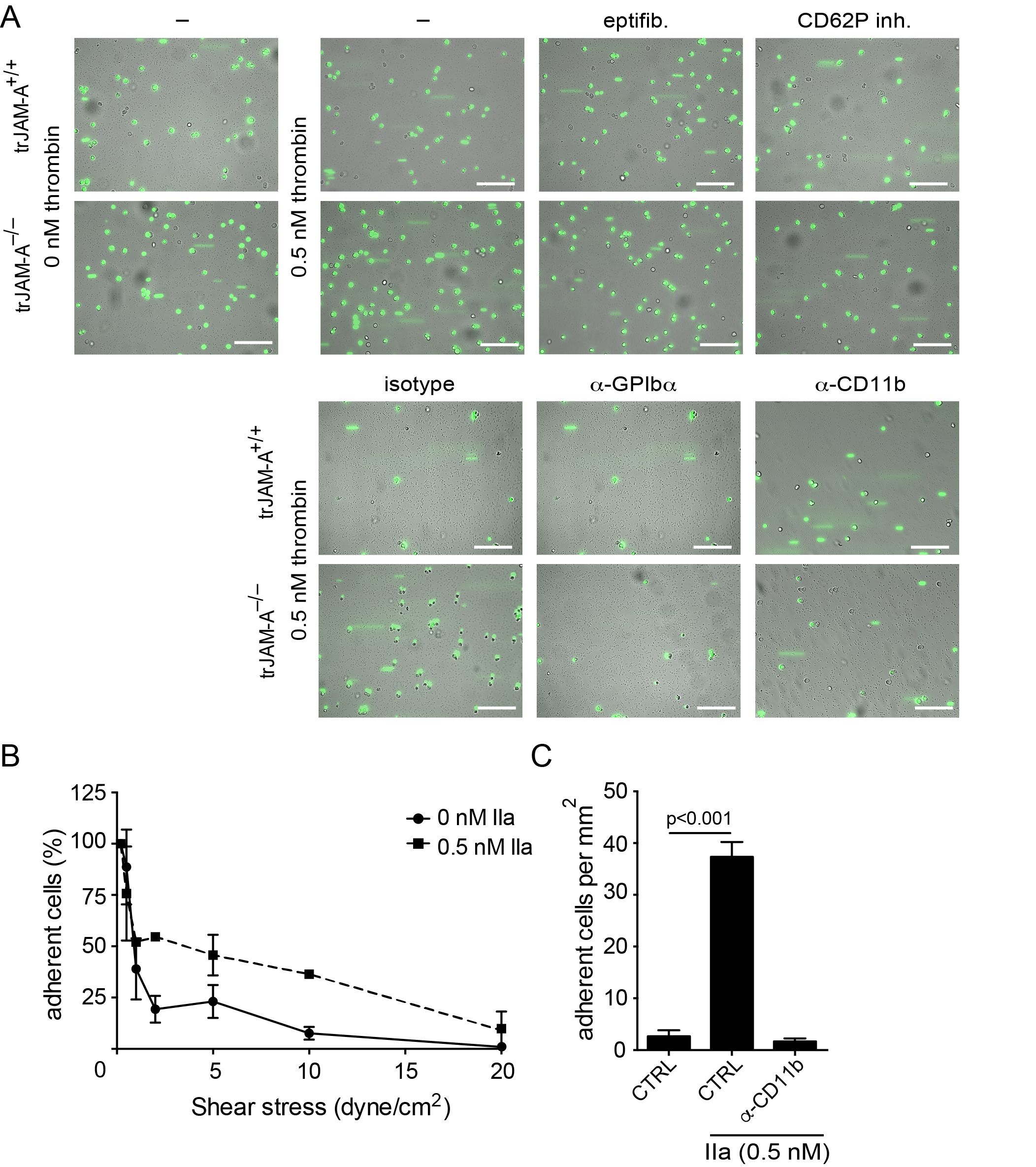


Figure S1. **Adhesion of monocytic cells to immobilized platelets.**

A. Representative micrographs of stable interactions of RAW264.7 cells with platelets from JAM-A^+/+^ and JAM-A^–/–^ mice immobilized on collagen-coated glass slides under flow conditions, without or with thrombin activation (IIa, 0.5 nM) in the presence of indicated inhibitors. Fluorescence and brightfield channels were mixed. Scale bar: 100 µm. B. Cell adhesion expressed as percentage of initially adherent cells under incrementally increased shear stress (dyne/cm^2^) on immobilized platelets without or with thrombin activation (IIa, 0.5 nM). C. Adherent cells (%) on thrombin-activated platelets at a shear stress of 10 dyne/cm^2^ in the presence of blocking anti-CD11b antibodies. P values were calculated by one-way ANOVA with Tukey's post test (n=3, mean±SEM).
