# Supplementary material for: Deletion of junctional adhesion molecule A from platelets increases early‐stage neointima formation after wire injury in hyperlipidemic mice
Source: J Cell Mol Med. 2017 Feb 17;21(8):1523–31. doi: 10.1111/jcmm.13083 (PMC5542900; doi:10.1111/jcmm.13083)
Supplement: Supplementary file 2 — Figure S2 Neointima area and composition in trJAM‐A+/+ apoe−/− and trJAM‐A−/− apoe−/− mice, 4 weeks after wire‐injury [file JCMM-21-1523-s002.docx]

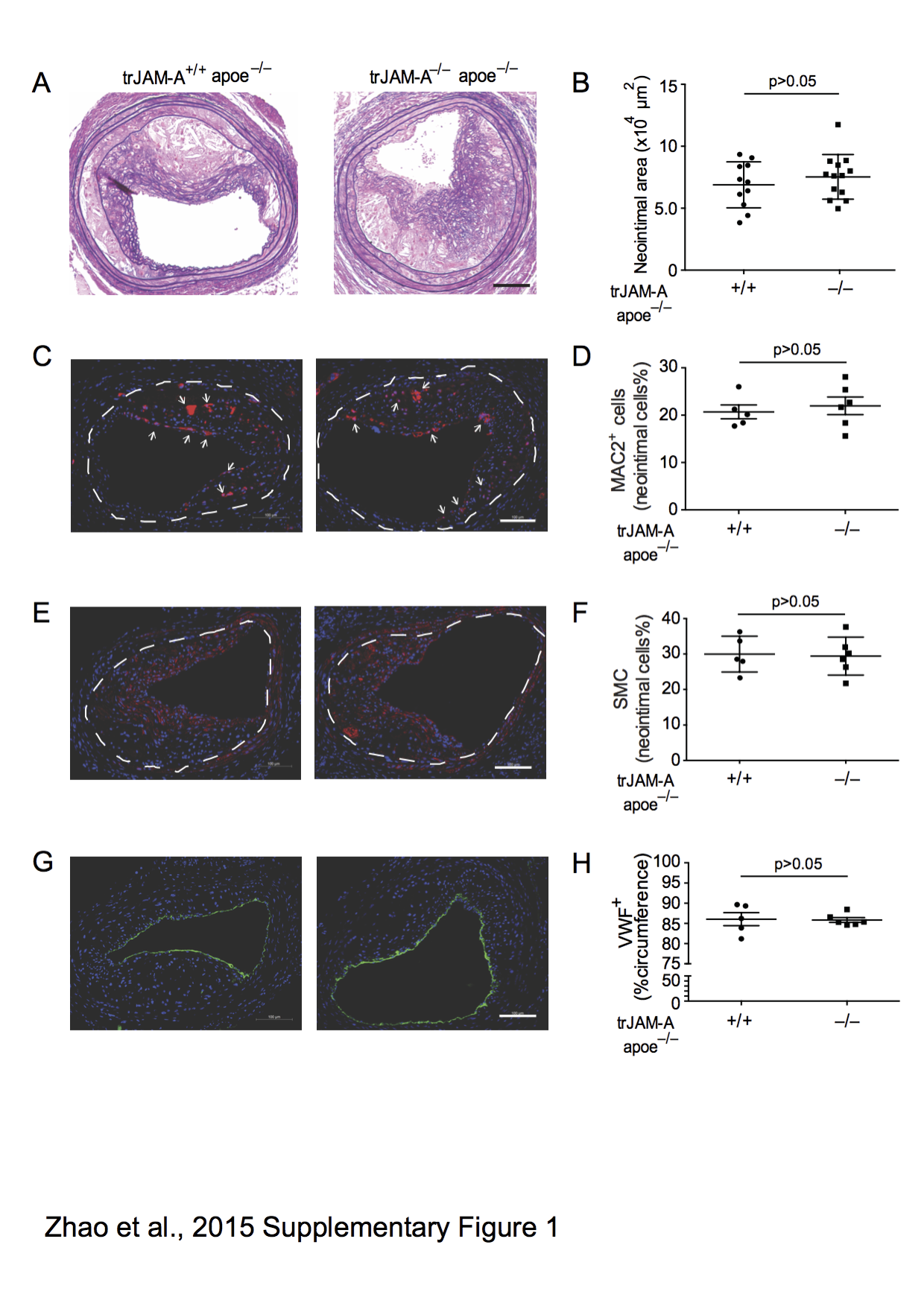


Figure S2. **Neointima area and composition in trJAM-A^+/+^ apoe^–/–^ and trJAM-A^–/–^ apoe^–/–^ mice, 4 weeks after wire-injury.** Neointima 4 weeks after wire-injury was visualized by EVG staining **(A)** and quantification **(B)** of neointimal area (µm^2^). Immunofluorescence of neointimal macrophages (**C**, MAC2^+^, green), SMC (**E**, α-Actin^+^, red) and their quantification (**D**, MAC2**, F**, SMC). Endothelium was visualized by staining of vWF (green) **(G)** and length of vWF^+^ lining was expressed as percentage of luminal circumference **(H)**. P values were calculated by unpaired t-test (B, n=11-13 , D,E,F n=5-6). Neointima areas were demarcated with dashed lines. Nuclei were stained with DAPI (blue). Scale bar: 100µm.
